# Anti-CD166/4-1BB chimeric antigen receptor T cell therapy for the treatment of osteosarcoma

**DOI:** 10.1186/s13046-019-1147-6

**Published:** 2019-04-17

**Authors:** Yitian Wang, Wei Yu, Jian Zhu, Junjie Wang, Kaishun Xia, Chengzhen Liang, Huimin Tao

**Affiliations:** 1 Department of Orthopedics, 2nd Affiliated Hospital, School of Medicine, Zhejiang University, #88 Jie Fang Road, Hangzhou, 310009 Zhejiang People’s Republic of China; 2 Orthopedics Research Institute of Zhejiang University, No. 88, Jiefang Road, Hangzhou, 310009 China

**Keywords:** CAR-T, CD166, 4-1BB, Osteosarcoma, Immunotherapy

## Abstract

**Background:**

Chimeric antigen receptor (CAR)-engineered T cells have displayed outstanding performance in the treatment of patients with hematological malignancies. However, their efficacy against solid tumors has been largely limited.

**Methods:**

In this study, human osteosarcoma cell lines were prepared, flow cytometry using antibodies against CD166 was performed on different cell samples. CD166-specific T cells were obtained by viral gene transfer of corresponding DNA plasmids and selectively expanded using IL-2 and IL-15. The ability of CD166.BBζ CAR-T cells to kill CD166^+^ osteosarcoma cells was evaluated in vitro and in vivo.

**Results:**

CD166 was selectively expressed on four different human osteosarcoma cell lines, indicating its role as the novel target for CAR-T cell therapy. CD166.BBζ CAR-T cells killed osteosarcoma cell lines in vitro; the cytotoxicity correlated with the level of CD166 expression on the tumor cells. Intravenous injection of CD166.BBζ CAR-T cells into mice resulted in the regression of the tumor with no obvious toxicity.

**Conclusions:**

Together, the data suggest that CD166.BBζ CAR-T cells may serve as a new therapeutic strategy in the future clinical practice for the treatment of osteosarcoma.

**Electronic supplementary material:**

The online version of this article (10.1186/s13046-019-1147-6) contains supplementary material, which is available to authorized users.

## Background

Osteosarcoma (OS) is the primary malignant bone tumor affecting children and adolescents. It is inclined to occur in the metaphysis of long bones, including distal femur and proximal tibia [1]. The treatment strategy for OS has advanced from amputation to the current neoadjuvant chemotherapy, surgery and the subsequent adjuvant chemotherapy. Despite the introduction of various chemotherapy regimens, the overall survival rate for advanced or recurrent OS patients still remains low [2, 3]. Therefore, novel treatment regimens with improved therapeutic benefits need to be investigated.

Cellular immunotherapy is the emerging strategy that is of great interest in oncology. Adoptive cell transfer (ACT), in particular, is a promising regimen attributed to the recent success of CD19-chimeric antigen receptor (CAR)-T cells against acute lymphoblastic leukemia [4–6]. Specifically, the genetic addition of CARs enables T cells to target tumor cells in a major histocompatibility complex (MHC)-unrestricted manner [7]. Despite its consistently remarkable antitumor activity against hematological malignancies, CAR-T therapy in the treatment of solid tumors remains challenging mainly due to the lack of proper tumor associated antigen (TAA) [8].

Activated leukocyte cell adhesion molecule (ALCAM, CD166) is a 105 kDa trans-membrane glycoprotein that belongs to the immunoglobulin superfamily. The binding of ALCAM specifically to CD6 mediates the interaction between adjacent cells [9]. ALCAM is considered to function in a various biological activities including neuronal outgrowth, hematopoiesis and inflammatory responses [10]. Previous studies have demonstrated its association with tumorigenesis of many malignancies, including breast cancer, prostate cancer, melanoma and OS [11–14]. ALCAM can also function as the therapeutic target using anti-ALCAM monoclonal antibody, conjugated to the nanoparticles to eliminate prostate cancer and OS cells [11, 15]. All these evidence qualifies ALCAM as the promising candidate to target OS in the adoptive cell immunotherapy.

In the present work, we found that ALCAM was expressed in four different human OS cell lines at levels ranging from 36.9 to 96.7%. The generated CD166 CAR-T cells incorporated with 4-1BB demonstrated their cytotoxic activity against OS in vitro and in vivo, the cytotoxicity of which correlated well with the expression levels of CD166. Our results support future investigations of CD166 CAR-T cells and rational combinations with other immunotherapy for the treatment of OS and other CD166 positive malignancies.

## Methods

### Cell lines and cell culture

The human osteosarcoma lines MNNG/HOS, U2OS, MG-63 and Saos-2, normal human osteoblasts hFOB 1.19, normal human fetal lung fibroblasts HFL1 were obtained from Cell Bank of Shanghai Institute of Biochemistry and Cell Biology, Chinese Academy of Sciences (Shanghai, China). Normal human hepatocytes HL-7702 were obtained from Procell (Wuhan, China). Their identity was verified by short tandem repeat analysis. MG-63, MNNG/HOS, Saos2 cells were cultured in Dulbecco’s modified Eagle’s medium (Gibco, Rockville, MD, USA), U2OS cells and HL-7702 cells were cultured in RPMI 1640 medium (Gibco), HFL1 cells were cultured in F-12 K medium (ATCC), hFOB 1.19 cells were cultured in DMEM/F-12 medium (Gibco). All the medium was supplemented with 10% fetal bovine serum (Invitrogen, Carlsbad, CA, USA) and 100 μg/mL streptomycin–penicillin. All the cell lines were maintained at 37 °C in a humidified incubator containing 5% CO_2_. The cells were always passaged when they approximately reached 80% confluence.

### Construction of the anti-CD166 CAR

The chimeric CD166/CAR is composed of CD166 scFv and a 4-1BB-CD3ζ expression cassette that was designed and synthesized by the GeneChem Biotechnology Company (Shanghai, China), as is shown in Fig. 2a. The CD166 scFv was derived from a high-affinity monoclonal antibody. The 4-1BB-CD3ζ expression cassette contains the hinge and transmembrane (TM) region of CD8α. CD166 scFv and 4-1BB-CD3ζ were connected in-frame by overlap PCR. The generated CD166/CAR was verified by DNA sequencing and cloned into the BamHI sites of a lentiviral vector (Genechem Biotechnology, China); the resultant product was named CD166.BBζ CAR. The intracellular domain of the CARs has the self-cleaving 2A peptide connected to a GFP green fluorescent label. The sequences of all PCR primers are available upon request.

### Transduction of lentiviral CD166/CAR

Peripheral blood mononuclear cells (PBMCs) were isolated using the Ficoll density gradient centrifugation methods from whole blood of healthy volunteer donors. T cells were transfected with an Easy-T kit (GeneChem Biotechnology, China). Briefly, isolated T cells/PBMCs were activated on a plate pre-coated with S buffer (EASY-T cell infection activation kit, catalog no. LCR6018, GeneChem) at a concentration of 0.7 × 10^6^ cells/mL in complete RPMI 1640 medium (Gibco) supplemented with 10% fetal bovine serum (Invitrogen, Carlsbad, CA, USA), 50 IU/mL IL-15 and 200 IU/mL IL-2 (PeproTech). Two days later, the stimulated T cells were resuspended at 0.5 × 10^6^ cells/mL with the Trans B buffer (EASY-T cell infection activation kit, catalog no. LCR6018, GeneChem). CAR-encoding lentivirus (CD166.BBζ CAR) was thawed and added into the cells resuspension solution (virus titer: 5 × 10^8^ TU/mL, MOI = 5). The cells were seeded onto plates that had been coated for 16 h with Trans A buffer (EASY-T cell infection activation kit, catalog no. LCR6018, GeneChem). After 24 h transduction, equal volume of fresh medium supplemented with IL-2 (300 IU/mL) and IL-15 (100 IU/mL) was added into the plates. T cells were then fed with fresh media every 2 days and were used within 28 days of expansion in all experiments.

### Cytotoxicity assays

The cytotoxic activity of the CD166.BBζ CAR and non-transduced T cells (NTD) was evaluated using the CytoTox 96® Non-Radioactive Cytotoxicity Assay (Promega). Lactate dehydrogenase (LDH) release was evaluated after 4 h in the supernatant with effector-to-target (E:T) ratios of 20:1, 10:1 and 1:1.

### Cytokine release detection

CD166.BBζ CAR and non-transduced T cells were plated at 1 × 10^6^ cells per well on a 96-well plate at a 1:1 ratio with Saos-2 and U2OS cells. Interleukin-2 (IL-2), interleukin-4 (IL-4), interleukin-6 (IL-6), interleukin-10 (IL-10), tumor necrosis factor (TNF-α), and interferon-γ (IFN-γ) cytokine release after 24 h of culture was measured using the cytometric bead array (CBA) human Th1/Th2 cytokine kit (BD Bioscience).

### Flow cytometry

FITC-, PE-, perCP/cy5.5-, APC-, AF700- and PE/Cy7- conjugated anti-CD3, CD4, CD8, CD56, CD44, CD62L, CD86, CD197, CD25, PD-1, CD45RO monoclonal antibodies were used to stain lymphocytes (all from Biolegend), whereas the anti-CD166 mAb was used to label osteosarcoma cells.

### Immunochemistry

For the detection of T cells infiltration into the tumors, animals were sacrificed and tumors were harvested embedded in OCT medium, snap frozen or formalin fixed and paraffin-embedded. The specimens were cut into serial sections of 6 μm. To show the intratumoral T cells, staining of anti-CD3 (Abcam) was performed on consecutive tissue sections. Images were obtained using confocal microscope (Nikon A1, Japan) or optical microscopes, respectively.

### The in vivo antitumor activity of CD166/CAR-T cells in an orthotopic osteosarcoma model

7-week-old NOD/SCID (non-obese diabetic and severe combined immunodeficiency) mice (6–8 weeks old; 18–22 g) were obtained from Experimental Animal Center of the Zhejiang Chinese Medical University and maintained under SPF grade conditions and supplied with sterilized food and water. The use of all mice in this study were approved by the Animal Care and Use Committee of Zhejiang University, China. Saos-2 cells were transfected with luciferase (Saos2-fLuc) for in vivo imaging. The orthotopic osteosarcoma model was established according to the previous studies [16]. Briefly, the mouse was anesthetized by isoflurane, and a 30G needle was inserted to the proximal tibia through the cortex of the anterior tuberosity. Then Saos2-fLuc cells (5 × 10^6^ in 25 μL PBS) were injected slowly into the medullary cavity using Hamilton Syringe fitted with a 26G needle. Mice were randomly separated into three groups (five mice in each group). After 7 days, tumors in different groups reached the same volume, which was confirmed by In Vivo Imaging System (IVIS) (Lumina Series III, Caliper life sciences), treatments of each group were then set as follows: (1) untreated mice, receiving 100 μL PBS, (2) non-transduced-T cells (1 × 10^7^) in 100 μL PBS, (3) CD166.BBζ CAR-T cells (1 × 10^7^) in 100 μL PBS. Tumor progression was confirmed by the measurement of bioluminescence intensity using the IVIS for up to 28 days.

### Statistical analysis

All the data were analyzed using the SPSS software (version 16.0, SPSS, Chicago, IL, USA) and presented as mean ± SD. The statistical differences were detected by Student’s *t*-test, one-way analysis of variance (ANOVA) with Dunnett’s test or two-way ANOVA analysis. *p* < 0.05 was considered to be statistically significant.

## Results

### Expression of CD166 in osteosarcoma cell lines

**Fig. 1:**
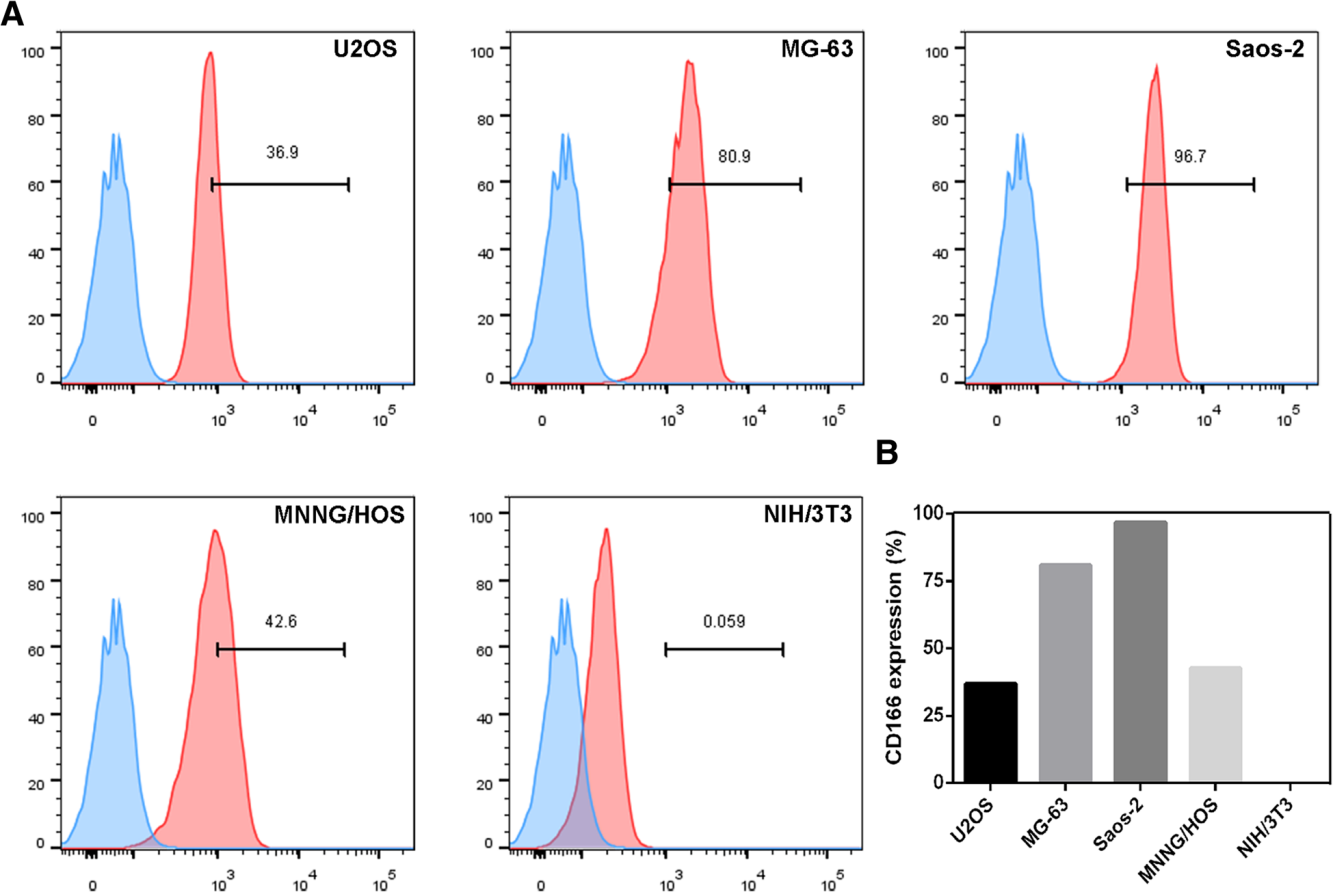
Expression of CD166 antigen on human osteosarcoma cell lines. **a**. The expression of CD166 on four human osteosarcoma cell lines was evaluated by FACS analysis. Saos-2, MG63 cell lines exhibited CD166 expression at high levels, MNNG/HOS, U2OS cell lines exhibited CD166 expression at low levels, respectively (red histograms). For NIH/3 T3 cell lines, CD166 was undetectable. A CD166 isotype antibody was used as a negative control for the detection of CD166 expression (blue histograms). **b**. The histogram of GD2 expression on NIH/3 T3 and human osteosarcoma cell lines

As reported by literature, a relatively high expression of CD166 was found both in primary OS specimens and tumor-derived cell lines [11]. Then, we re-analyzed microarray data from GEO dataset (Access id: E-MEXP-3628) and found that CD166 expression significantly increased in OS tumor tissues when compared with adjacent ones (Additional file 1: Figure S1). We also analyzed data of OS patients from ArrayExpress (Access id: GSE21257) and found that OS patients who developed metastases in five years had significantly higher expression of CD166 than the ones without metastases (Additional file 1: Figure S2). Therefore, we speculated that osteosarcoma-bearing patients may benefit from the CD166-specific CAR-T cells therapy. Subsequently, we examined the CD166 expression in a panel of four human osteosarcoma cell lines using flow cytometry (Fig. 1). The results confirmed the relatively high level of CD166 expression on the surface of osteosarcoma cell lines, which varied between 36.9 to 96.7%. As a contrast, no CD166 expression was found on the surface of the fibroblast cell line NIH/3 T3.

### CD166.BBζ CAR-T cells were successfully modified by lentiviral CD166/CAR

**Fig. 2:**
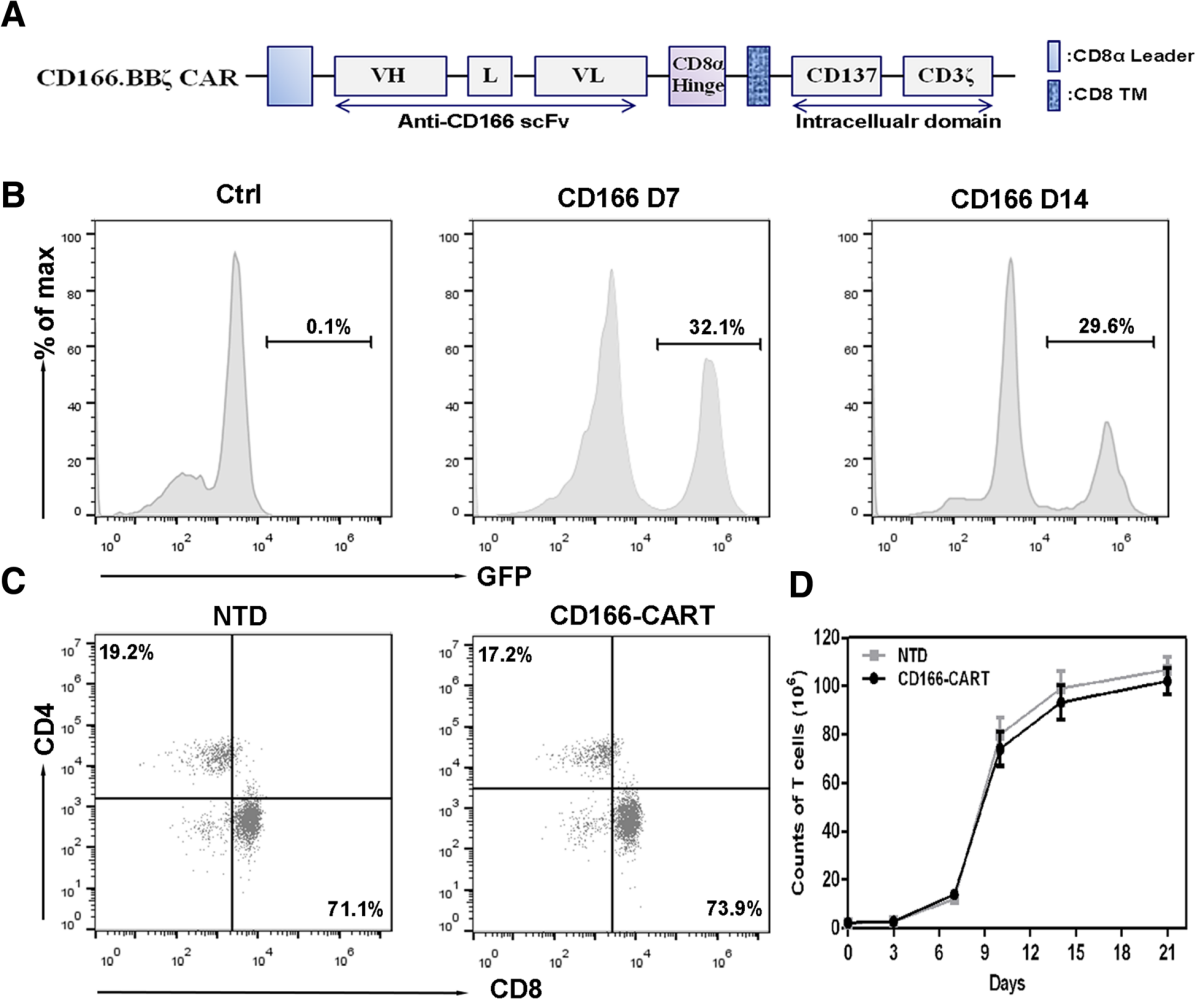
Generation of CD166-CART cells in vitro. **a**. Schematic representation of CD166-based CAR constructs containing the CD3ζ cytosolic domain in combination with the CD137 costimulatory module (CD166.BBζ CAR). VL: variable L chain, L linker, VH: variable H chain, and TM: transmembrane region. **b**. A representative of CD166-CARs expression on human T cells transduced with lentivirus was analyzed using flow cytometry, which detected GFP expression at day 7 and 14. **c**. The expression of CD166-CARs in CD4^+^ and CD8^+^ T lymphocytes of non-transduced T cell group and CD166-CART group after the transduction. **d**. The expansion of different T cells in vitro from day 0 to day 21. Results represent mean ± SD of three individual experiments

In order to generate CD166 CAR-expressed T cells in vitro, we first constructed the lentivirus vectors containing the sequence encoding the anti-CD166 scFv. The CD166-directed CAR expression was composed of an anti-CD166 scFv fused to a CD8α hinge and transmembrane region and the intracellular signaling domains of human 4-1BB and CD3ζ motif in tandem (Fig. 2a). The surface expression of CD166/CAR on the T cells was measured by flow cytometry through the detection of GFP. As shown in Fig. 2b, flow cytometric analysis confirms that the frequency of CAR expression was 32.1% for CD166 CAR, which was stable from day 7 to day 14 (29.6%) without significant difference. 7 days after the lentiviral CD166/CAR transduction, the generated CAR T cells were > 98% CD3-positive T cells, which shared nearly the same ratio of CD4- and CD8- positive T cell subsets with the non-transduced T cells (Fig. 2c). During our culture process, T cells started to expand at day 3 and continued to proliferate until day 21, and 40- to 50 folds of T cell reproducible expansion can be managed at day 14 (Fig. 2d). Together, these results confirmed our successful construction of CD166.BBζ CAR-T cells and robust expansion of transduced T cells from healthy donors.

### Phenotypic characterization of CD166.BBζ CAR-T cells in vitro

**Fig. 3:**
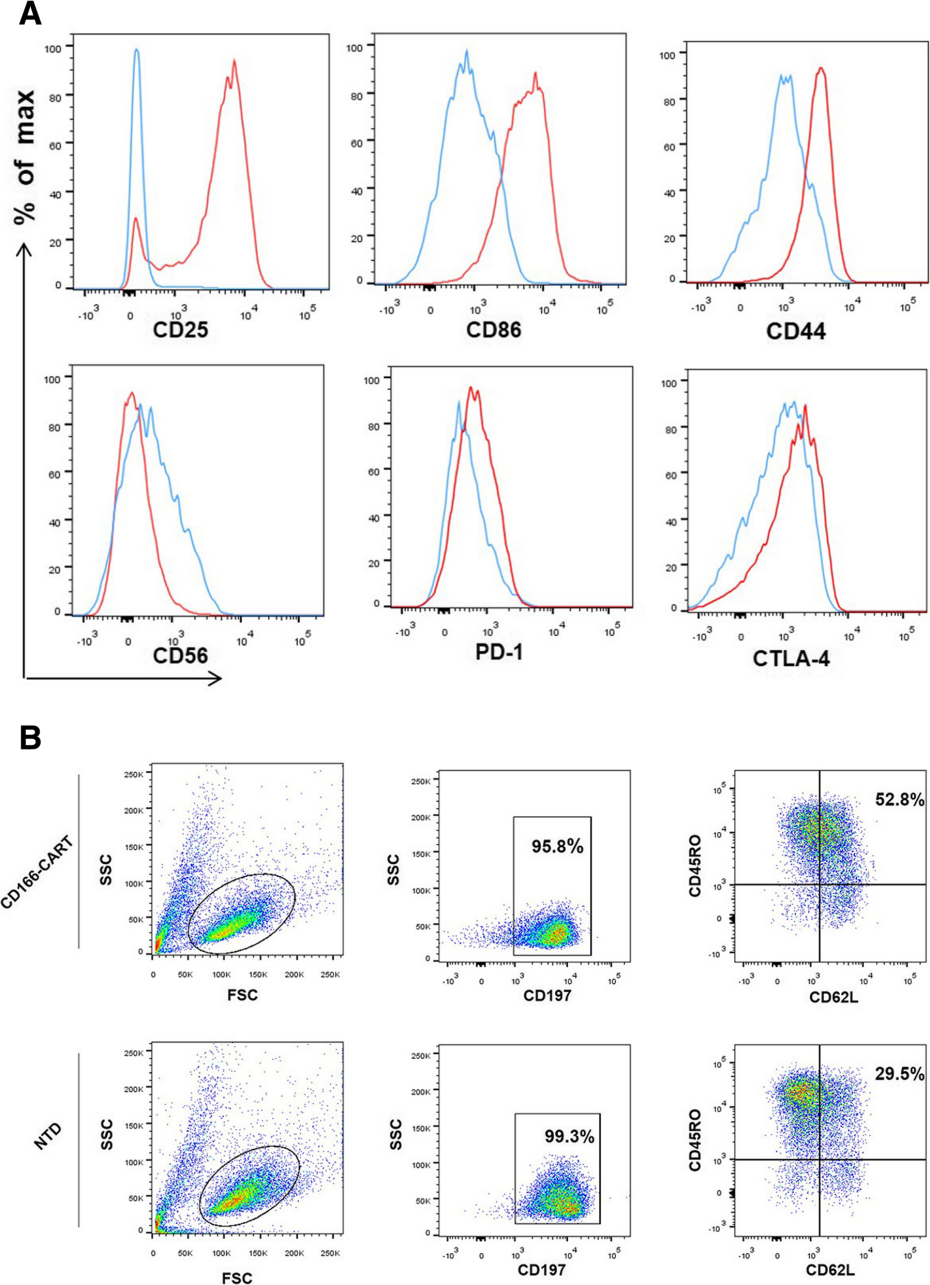
Phenotypic analysis of CD166.BBζ CAR-T cells in vitro. **a**. Flow cytometry comparison of the common surface phenotypes of CD166.BBζ CAR-T cells (red line) at day 14 of culture with freshly isolated T cells (blue line). Histogram overlays show 6 markers related to lymphocyte activation, differentiation, migration, adhesion, and exhaustion. **b**. Central memory T phenotypes of CD166.BBζ CAR-T cells and non-transduced T cells were evaluated by flow cytometry

In order to better define the characteristics of CAR-T cells after transduction, we then adopted phenotypic analysis. The CAR-T cells were compared at indicated time points during the culture process (day 1 and day 14). As shown in Fig. 3a, significant upregulation of activation marker CD25 and costimulatory molecules CD86 were observed, indicating the improved reproducible potentials of CAR-T cells. Besides, expression changes of cell adhesion-associated molecules were also detected, such as CD44 and CD56. What’s more, the exhaustion and inhibitory markers (PD-1, CTLA-4) of CD166.BBζ CAR-T cells were upregulated 14 days after initial activation. As shown in Fig. 3b and Additional file 1: Figure S3, proper fraction of CD166.BBζ CAR-T cells expressed the indicated central memory T phenotypes (CD45RO^+^CD62L^+^CCR7^+^), which was significantly higher than the corresponding population of non-transduced T cells.

### CD166.BBζ CAR-T cells exhibited specific and potent cytotoxicity against human OS cells

**Fig. 4:**
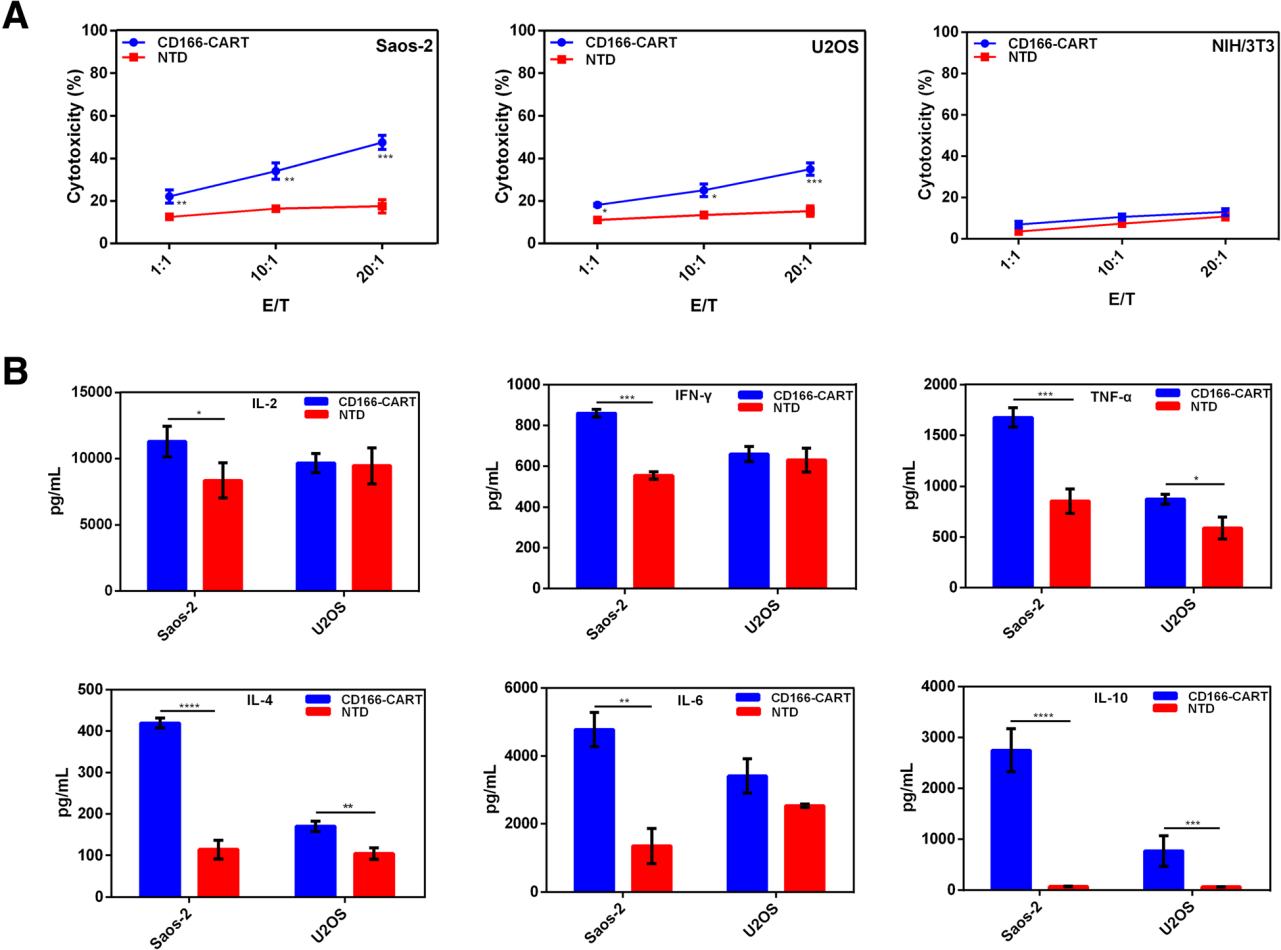
Functional analysis of CD166.BBζ CAR-T cells in vitro. **a**. Cytotoxic activity of CD166.BBζ CAR-T cells. We used the LDH release assay to evaluate the cytotoxic activity of CD166.BBζ CAR-T cells and non-transduced T cells at different E/T ratios (effector/ target cells). **b**. Th1/ Th2 cytokine release of CD166.BBζ CAR-T cells. Culture supernatant was collected 24 h later, and the production of IL-2, TNF-α, IFN-γ, IL-4, IL-6, IL-10 were measured using a CBA assay. The results are presented as the mean ± SD from experiments that were performed in triplicate (*P < 0.05, **P < 0.01, ***P < 0.001, **** *P* < 0.0001)

The LDH release assay was adopted to verify the specific lytic function of CD166.BBζ CAR-T cells against two human osteosarcoma cell lines with varying CD166 expression levels. After 4 h of co-culture, CD166.BBζ CAR-T cells efficiently lysed CD166^high^ Saos-2 cells, but not CD166-negative NIH/3 T3 cells, while the cytotoxicity against CD166^low^ U2OS cells was comparatively modest (Fig. 4a). This confirmed that the cytotoxicity of CD166.BBζ CAR-T cells correlated with the level of CD166 expression. Besides, the enhanced cytotoxicity of CD166.BBζ CAR-T cells against OS cells was accompanied with the elevated E:T ratio. Collectively, these results demonstrate the specificity and potency of CD166.BBζ CAR-T cells against CD166-positive osteosarcoma cells.

Human TH1/TH2 cytokine CBA kit was adopted to determine the cytokine release from CD166.BBζ CAR-T cells when co-cultured with targets of different CD166 expression. Consequently, a large amount of TNF-α, IFN-γ was released by CD166.BBζ CAR-T cells and was associated with the quantity of CD166 expression (Fig. 4b). In contrast, the release of IL-2 from CD166.BBζ CAR-T cells was quite modest when compared with the non-transduced T cell group. To note, CAR-T cells also produced considerable amount of Th2 cytokines, such as IL-4, IL-6 and IL-10. The expression of these cytokines also correlated with the CD166 expression level on different target cells.

### Adoptive transfer of CD166.BBζ CAR-T cells induced the regression of orthotopic OS in vivo

**Fig. 5:**
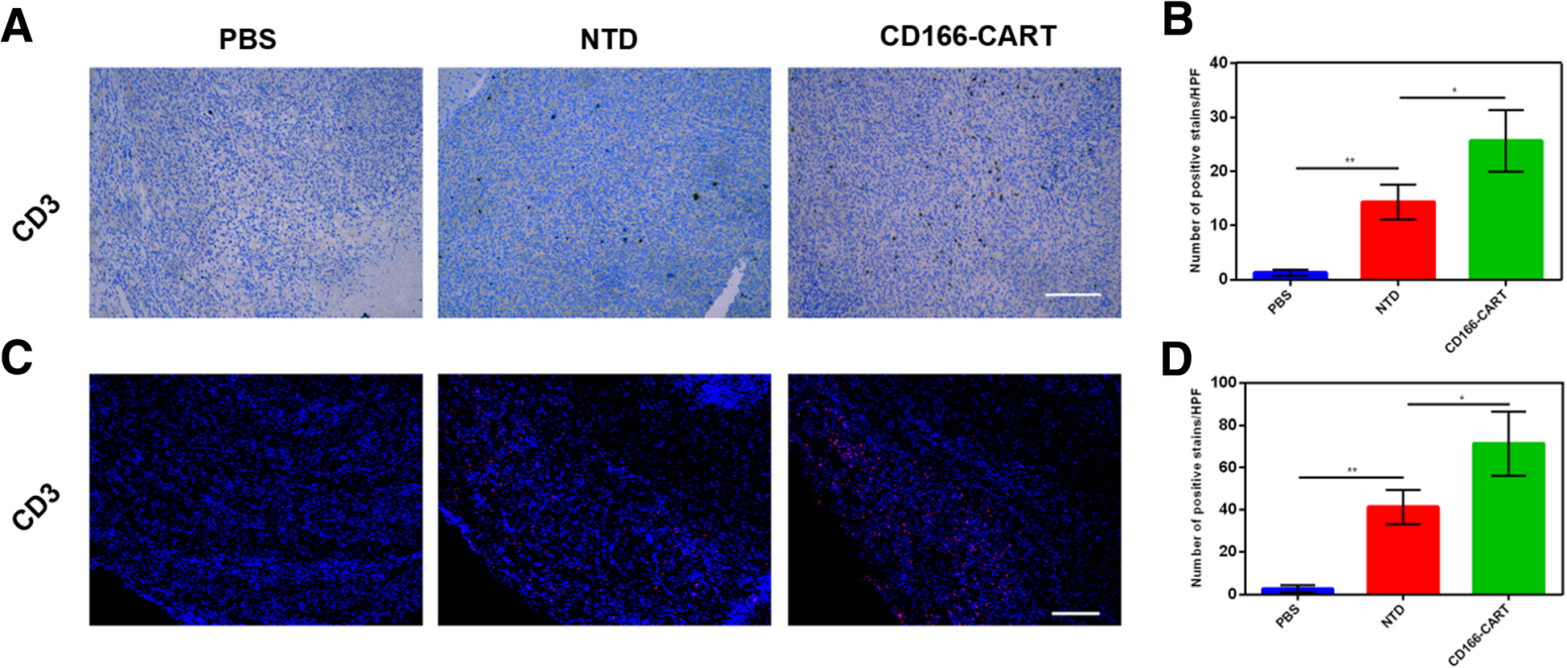
Tumor targeting ability of CD166.BBζ CAR-T cells was evaluated. Intratumoral T cells were detected by immunohistochemical assays (**a**, **b**) (shown in brown) and immunofluorescence (**c**, **d**) (shown in red), scale bar, 200 μm. The results are presented as the mean ± SD from experiments that were performed in triplicate (*P < 0.05, **P < 0.01)

To evaluate the efficacy of CD166.BBζ CAR-T cells against orthotopic OS in vivo, we injected Saos2-fLuc cells into the tibias of NOD/SCID mice, which led to the formation of palpable tumors within 7 days. Firstly, we investigated the ability of CAR-T cells to target the primary tumor. CD166.BBζ CAR-T cells and non-transduced T cells were *i.v.* injected once the tumor models were established. 48 h later, we sacrificed the mice and excised the tumors for analysis. Intratumoral T cells were detected by immunofluorescence and IHC assay using CD3 antibodies. As we can see in Fig. 5, the number of T cells found in the CD166.BBζ CAR-T cells group was significantly higher than that in the non-transduced T cells group.

**Fig. 6:**
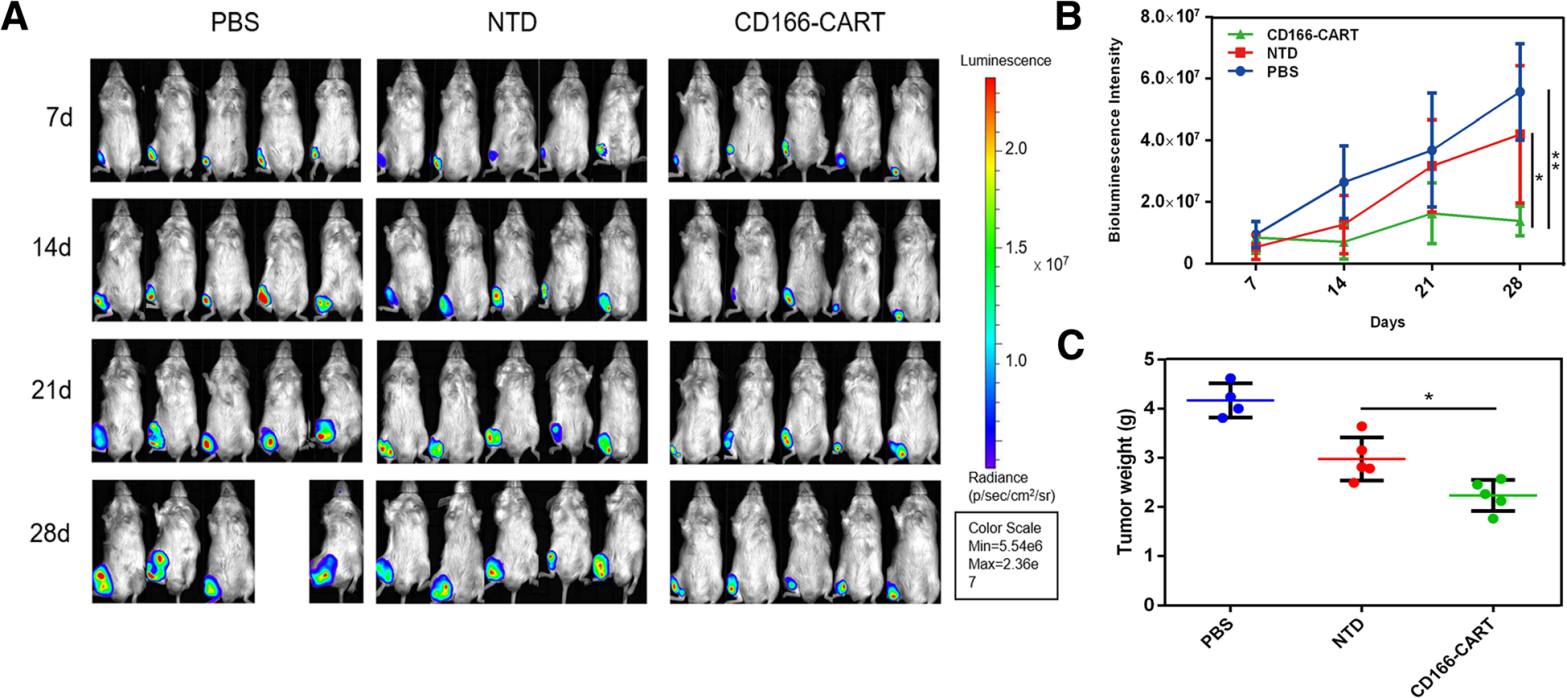
In vivo effects of human CD166.BBζ CAR-T cells on the inhibition of osteosarcoma cell xenografts. **a**. NOD/SCID mice were injected with Saos-2-fLuc cells for xenograft growth in mice and then injected with CD166.BBζ CAR-T, PBS (with the same volume) or non-transduced T cells *i.v.* on day 7, 14 and 21. IVIS imaging system was used to measure tumor growth. **b**. Bioluminescence intensities of osteosarcoma after adoptive T cell therapy were recorded. **c**. Osteosarcoma tumor weights from the mice treated in different groups at the end of the experiment. Results represent mean ± SD. *P < 0.05 and **P < 0.01 with T-test

After confirming the targeting ability of CAR-T cells, we then seek to investigate the efficacy of CD166.BBζ CAR-T cells against orthotopic OS in vivo. After the intra-tibia injection of Saos2-fLuc cells, the establishment of orthotopic osteosarcoma model was confirmed by bioluminescence imaging on day 7 (Fig. 6a). Since then, 1.0 × 10^7^ CAR T or NTD T cells (or PBS of the same volume) were *i.v.* injected into the tumor-bearing mice once a week for consecutive three weeks. The tumor xenografts were observed via IVIS for 21 days after the establishment of tumor models. As shown in Fig. 6a and b, the CD166.BBζ CAR-T cells could efficiently suppress tumor growth when compared to the control groups that received either NTD T cells or PBS. Besides, the examination of tumor weights as well as the tumor outlook after excision also confirmed the previous results (Fig. 6c, Additional file 1: Figure S4).

**Fig. 7:**
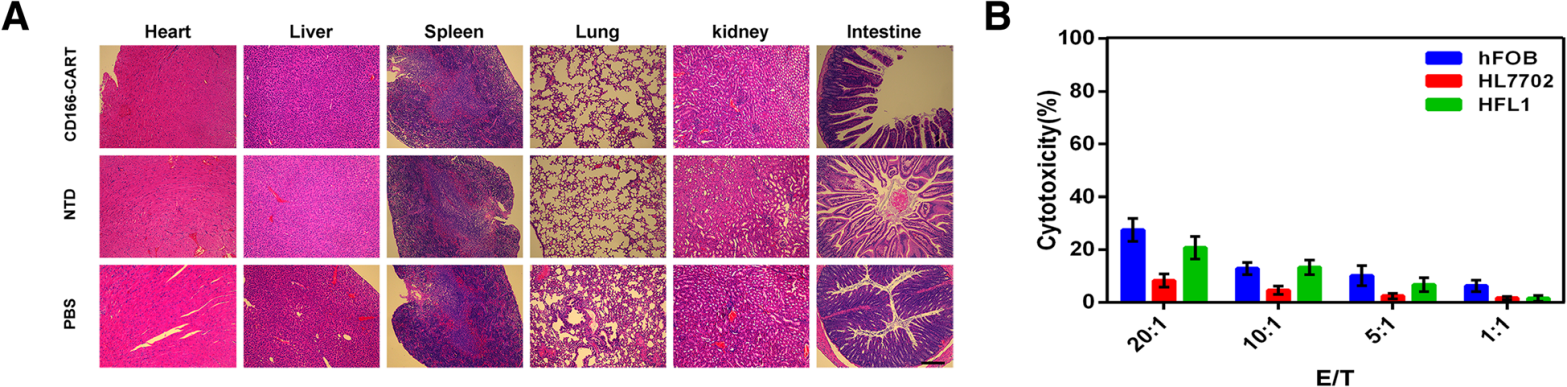
Safety evaluation of CAR-T therapy. **a**. H&E staining shows that there is no obvious off-target toxicity against mouse major organs. × 100 magnifications. Scale bar, 200 μm. **b**. CD166.BBζ CAR-T cells show no cytolytic activity against healthy HL-7702 cells. hFOB 1.19 and HFL1 cell lines are sensitive to CD166.BBζ CAR-T cells in vitro

Finally, in order to evaluate the potential toxicity of CD166.BBζ CAR-T cells, murine organs, including the lung, heart, liver, spleen, intestine and kidney, were excised and examined histologically. There were no detectable morphological changes caused by off-target toxicity after the infusion of CD166.BBζ CAR-T cells (Fig. 7a). To further verify that CD166.BBζ CAR-T cells have no cytotoxic activity against healthy tissues, hFOB 1.19, HL-7702 and HFL1 healthy cell lines were used as targets for in vitro lytic assays. No specific cytotoxic activity was observed against healthy HL-7702 cells. For HFL1 and hFOB 1.19 cell lines, CD166.BBζ CAR-T cells showed a low level of cytotoxicity (Fig. 7b). Expression of CD166 on healthy cells is shown in Additional file 1: Figure S5.

## Discussion

OS is an aggressive malignancy of bone characterized by surrounding calcified osteoid extracellular matrix and frequent lung metastases [17]. The prognosis of OS patients has achieved little improvement since the advent of chemotherapy. The 5-year overall survival remains dismal and stagnant for the last five decades [18]. Hence, there is an urgent need for the development of new therapeutic regimens. Several immunotherapies have been carried out in clinical trials against OS, including interferon α2b and muramyl tripeptide [19, 20]. However, these trials were plagued with different obstacles. ACT is another alternative strategy for the treatment of OS. Earlier efforts have been put on ACT for cytotoxic T lymphocytes and γδ T lymphocytes [21, 22], while recent studies focused mainly on genetic engineering of T lymphocytes with new antitumor specificities, including TCR-T Cells and CAR-T cells [23, 24]. Despite its favorable outcomes in treating melanoma and metastatic synovial cell sarcoma [24], the TCR-engineered T cell therapy still confronts many challenges, including low MHC complex binding affinity and decreased TCRs expression. In contrast, the single-chain variable fragment from the CAR-T cells enables them to bind and recognize targeting antigens in an MHC-independent way, thus overcoming barriers such as HLA downmodulation-related tumor escape and low epitope density-related T cell inactivation [25]. Due to its great advantages over traditional immunotherapies, CAR-T therapy has now been widely explored and adopted [26, 27].

Appropriate TAA selection is quite essential for the successful CAR-T therapy. Our results indicate that genetically modified T cells transduced to recognize CD166 may have therapeutic potential against orthotopic OS. Firstly, we demonstrated that CD166 was expressed by the OS cell lines with varying levels. CD166 has previously been identified in primary OS biopsy specimens with high frequency of expression [11]. Due to its vague role in the correlation between expression level and overall survival [13, 28], CD166 might have its limitation to serve as the prognostic marker in OS. Instead, it has the great potential to be adopted as the targeting molecule against OS. What’s more, the therapeutic potential of targeting CD166 has been shown by exploiting polymerized liposomal nanoparticles conjugated with corresponding antibody [11]. All these evidence taken together with our findings favor the idea of CD166 to serve as the promising targeting molecule for CAR-T therapy against OS.

It has been noted that intracellular signaling of T cells is essential for the activation of effector function and persistence of T cells. Meanwhile, just as most malignancies, the paucity of costimulatory molecules expression on the OS cells would make it insufficient for the complete activation of T cells while binding TAA via chimeric receptor. Previous studies have confirmed the advantages of 4-1BB (CD137) costimulation over CD28 for the improvement of T cell proliferation and reduction of exhaustion markers expression [29, 30]. Therefore, dual endodomains (4-1BB and CD3ζ) were introduced to form the second generation CAR. In the current work, we characterized the effects of CD166.BBζ CAR-T cells in the immunodeficient mice models of OS. Our findings showed that CD166.BBζ CAR-T cells were fully activated upon engagement with CD166-positive OS cells as demonstrated by their cytotoxicity and release of T helper type 1 (Th1) cytokines, which is crucial for the recruitment and maturation of antigen-presenting cells and improvement of cytotoxic T cell responses [31, 32]. Interestingly, despite 4-1BB’s contribution to the great levels of Th1 cytokines [33], Th2 distortive responses were also observed. Due to the possible suppressive immune responses from Th2-biased cytokine production, it is necessary to evaluate and improve the CAR construct before further application.

A major concern in immunotherapy research is the potential “on-target, off-tumor” toxicity [34]. CD166 expression is closely correlated with a wide variety of human cancers, including melanoma, head and neck squamous cell carcinoma, rectal cancer [35]. Meanwhile, it is also expressed on the surface of epithelial cells, fibroblasts and neurons [36–38]. It is worth noting that CD166-mediated interaction of antigen presenting cells with CD6 on T cells is considered to assign a role in T cell activation [39]. In the present study, the CD166-specific CAR-T therapy was safe and had no obvious off-tumor effect on mice as proved by histological examination. Nevertheless, we observed that CD166.BBζ CAR-T cells exhibited a low level of cytotoxicity against HFL1 (normal fetal lung) cells and hFOB 1.19 (normal osteoblasts) cells in vitro. For HFL1 cells, since placenta is believed to have immunosuppressive role, the cytotoxicity against normal fetal lung that we have observed might not be extrapolated to their behaviors in vivo [40]. As for hFOB 1.19 cells, since they were tranfected with vector pUCSVtsA58 and pSV2-neo, they may not be considered as an entirely “normal” cells, which could explain the cytotoxicity observed. All these data suggest that CD166.BBζ CAR-T cells therapy might be safe in humans. However, safety issues should be further evaluated in the immunocompetent mice models as human CD166.BBζ CAR-T cells were administered in NOD/SCID mice bearing human CD166^**+**^ OS in our studies. In addition, more strategies should be developed in the future work to reduce possible adverse events brought by CD166.BBζ CAR-T cells, including the engineering of multi-specific CAR-T cells and the optimization of therapeutic dose. Since there are other potential targets (such as Her-2) proposed for therapy against OS [23, 41], construction of bi-specific T cells with CD166 and Her-2 CARs may prove beneficial and limit their activation to tumor sites.

In the current work, OS progression in our mice models were partially inhibited following CD166.BBζ CAR-T cells administration. Herein, the efficacy to kill tumor cell lines in xenografts were modest compared with other CAR-T regimens [42, 43]. Therefore, it is necessary to exploit more regimens to improve anticancer efficacy in solid tumors, among which the combination of CAR-T therapy and other immunotherapies is quite promising. Emerging efforts seek to utilize success with immune checkpoint inhibitors in other malignancies by extending these strategies to OS. Despite the partial success in murine models, the results turned out to be disappointing in human trials [44, 45]. It has been suggested that the low mutational burden in OS makes it inadequate to generate adoptive immune responses [46]. In this context, it will be wise to combine these two immunotherapeutic regimens for the maximum of T cell activation in tumor lesions and their efficacy against solid tumors.

There are some limitations in our study that requires further acknowledgement. For instance, the NOD/SCID mice model are unable to fully simulate system reaction to human antigens like CD166, which might lead to the eruption of cytokine release syndrome and relevant toxicity. Thus, the safety issues related to off-tumor toxicity needs further studies to address.

## Conclusion

In summary, for the first time, the present study demonstrates that the administration of CD166.BBζ CAR-T cells is a viable approach for the treatment of OS. Our successful in vivo studies in mice prompt further investigation, particularly with regard to enhancing the efficacy of CD166.BBζ CAR-T cells and adopting safety modifications to circumvent potential adverse events in CAR-T therapy. This CD166-targeted T cell therapy represent a clinically appealing treatment strategy for OS patients with positive CD166 expression, thus providing the basis for additional explorations in the clinical application of immunotherapy against OS.

## Additional file

Additional file 1: **Figure S1.** CD166 expression was significantly increased in osteosarcoma tumor tissues when compared with the adjacent tissues of patients from GEO dataset E-MEXP-3628 (**P < 0.01). **Figure S2.** Osteosarcoma patients who developed metastases in five years had significantly higher expression of CD166 than the ones without metastases. Data from ArrayExpress(Access id: GSE21257), *P < 0.05. **Figure S3.** Central memory T phenotypic features of CD166. BBζ CAR-T and non-transduced T cells were evaluated by FACS analysis. Mean positive rates ± SD from three different T cell lines are shown (**P < 0.01). **Figure S4.** The orthotopic osteosarcoma from different groups were excised and imaged. **Figure S5.** Expression level of CD166 antigen on three normal human cell lines (red histograms). A CD166 isotype antibody was used as a negative control for the detection of CD166 expression (blue histograms). Percentage of positive cells are detailed in the histograms. (PDF 291 kb)

## Abbreviations

ACT: Adoptive cell transfer
ALCAM: Activated leukocyte cell adhesion molecule
CAR: Chimeric antigen receptor
CBA: Cytometric bead array
CTLA-4: Cytotoxic T cell-stimulating cytokine
FACS: Fluorescence-activated cell sorting
IFN-γ: Interferon-γ
IHC: Immunohistochemistry
IL-10: Interleukin-10
IL-2: Interleukin-2
IL-4: Interleukin-4
IL-6: Interleukin-6
IVIS: In Vivo Imaging System
MHC: Major histocompatibility complex
NTD: Non-transduced
OS: Osteosarcoma
PD-1: Programmed cell death protein 1
TAA: Tumor-associated antigen
TCRs: T cell receptors
TNF-α: Tumor necrosis factor-α
