# Supplementary material for: Anti-CD166/4-1BB chimeric antigen receptor T cell therapy for the treatment of osteosarcoma
Source: J Exp Clin Cancer Res. 2019 Apr 17;38:168. doi: 10.1186/s13046-019-1147-6 (PMC6471997; doi:10.1186/s13046-019-1147-6)
Supplement: Supplementary file 1 — Figure S1. CD166 expression was significantly increased in osteosarcoma tumor tissues when compared with the adjacent tissues of patients from GEO dataset E-MEXP-3628 (**P < 0.01). Figure S2. Osteosarcoma patients who developed metastases in five years had significantly higher expression of CD166 than the ones without metastases. Data from ArrayExpress(Access id: GSE21257), *P < 0.05. Figure S3. Central memory T phenotypic features of CD166. BBζ CAR-T and non-transduced T cells were evaluated by FACS analysis. Mean positive rates ± SD from three different T cell lines are shown (**P < 0.01). Figure S4. The orthotopic osteosarcoma from different groups were excised and imaged. Figure S5. Expression level of CD166 antigen on three normal human cell lines (red histograms). A CD166 isotype antibody was used as a negative control for the detection of CD166 expression (blue histograms). Percentage of positive cells are detailed in the histograms. (PDF 291 kb) [file 13046_2019_1147_MOESM1_ESM.pdf]

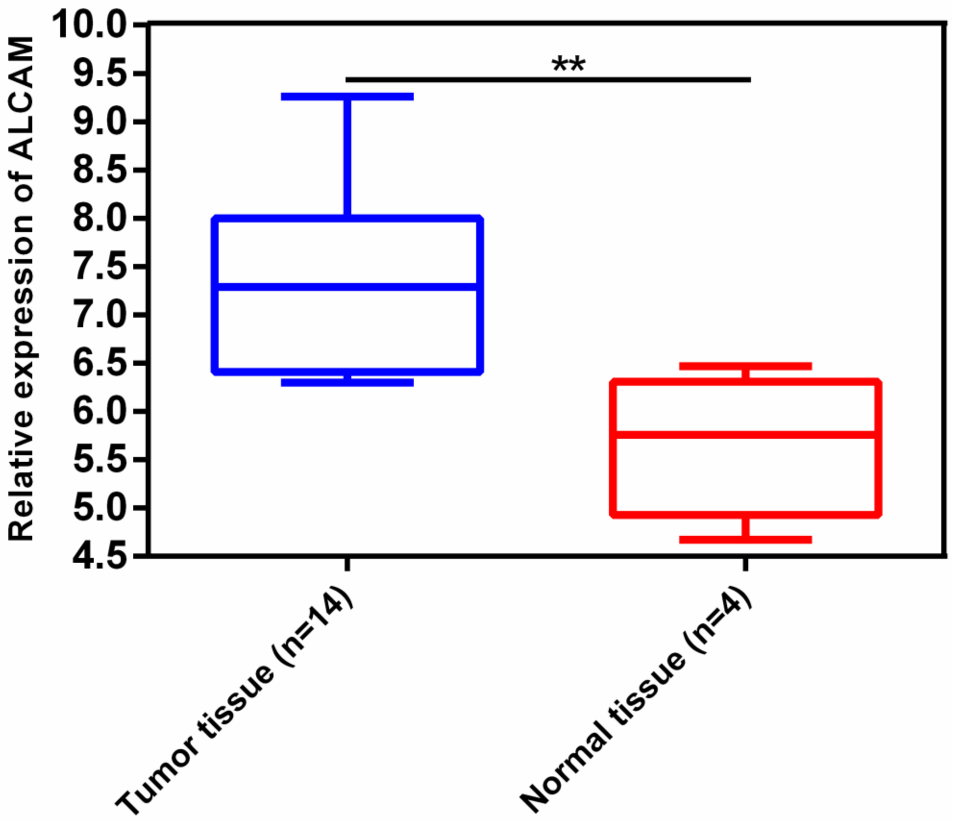

Figure S1. CD166 expression was significantly increased in osteosarcoma tumor tissues when compared with the adjacent tissues of patients from GEO dataset E-MEXP-3628 (**P < 0.01).

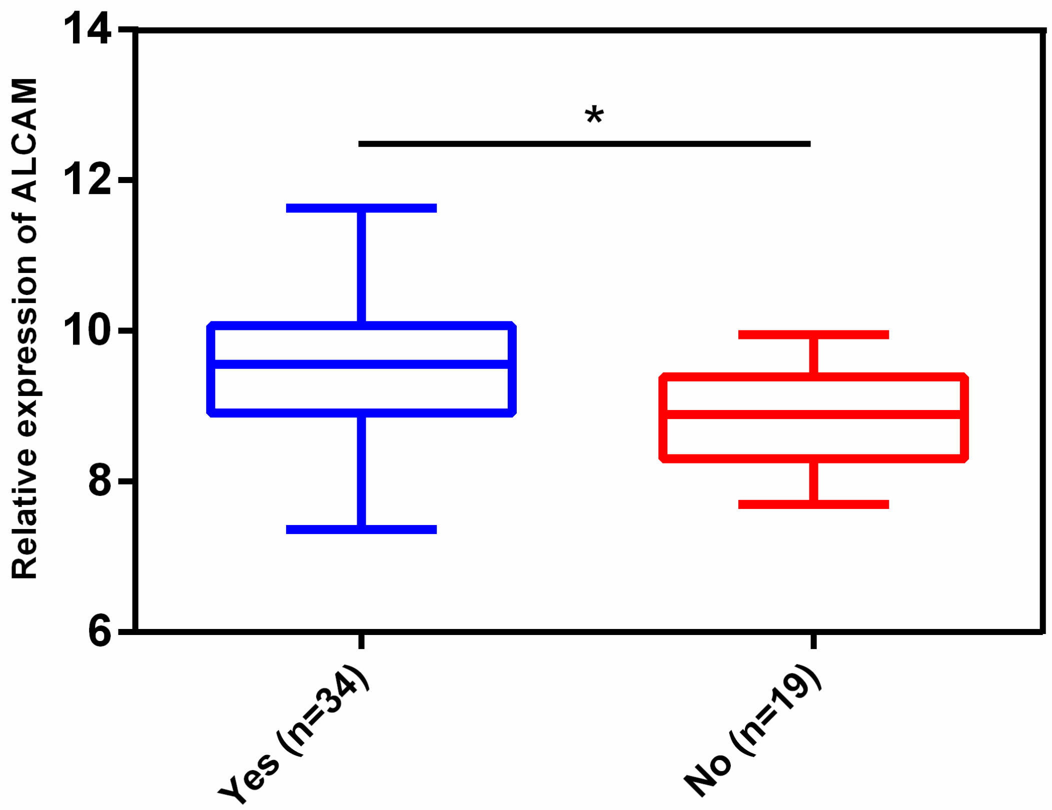

Figure S2. Osteosarcoma patients who developed metastases in five years had significantly higher expression of CD166 than the ones without metastases. Data from ArrayExpress (Access id: GSE21257), *$P < 0.05$.

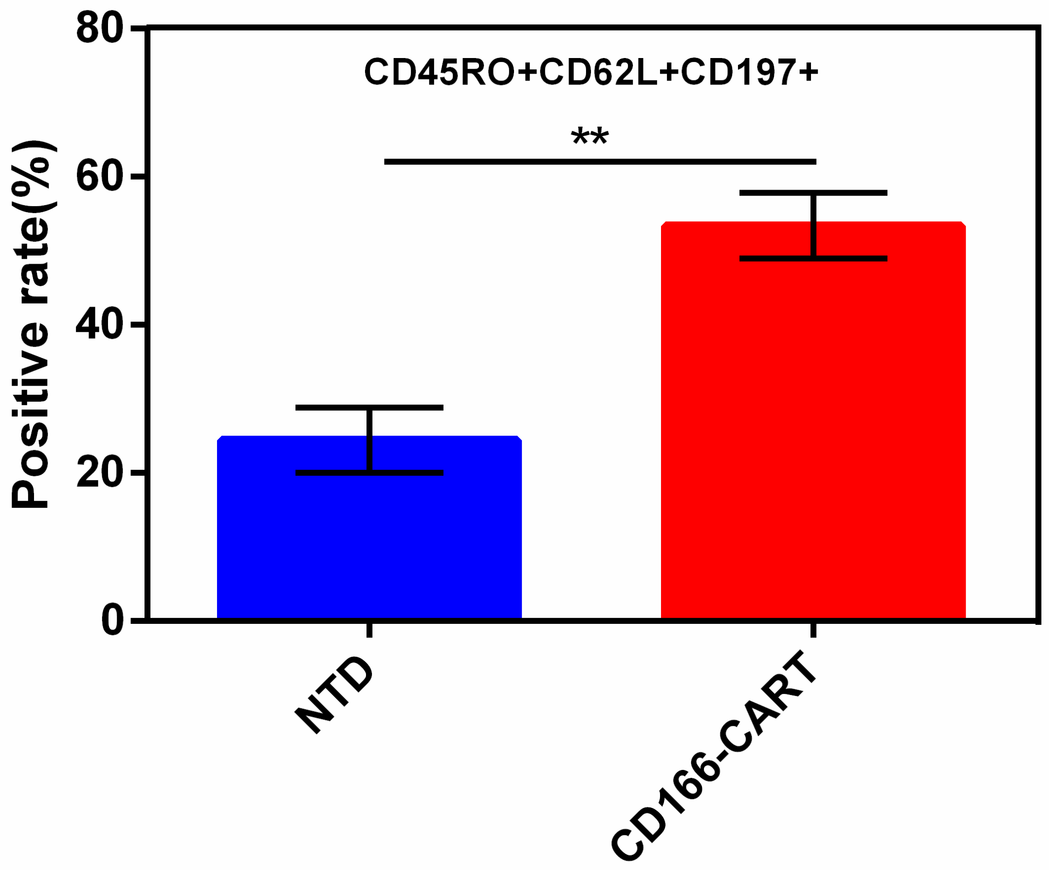

Figure S3. Central memory T phenotypic features of CD166.BBζ CAR-T and non-transduced T cells were evaluated by FACS analysis. Mean positive rates ± SD from three different T cell lines are shown (**$P < 0.01$) .

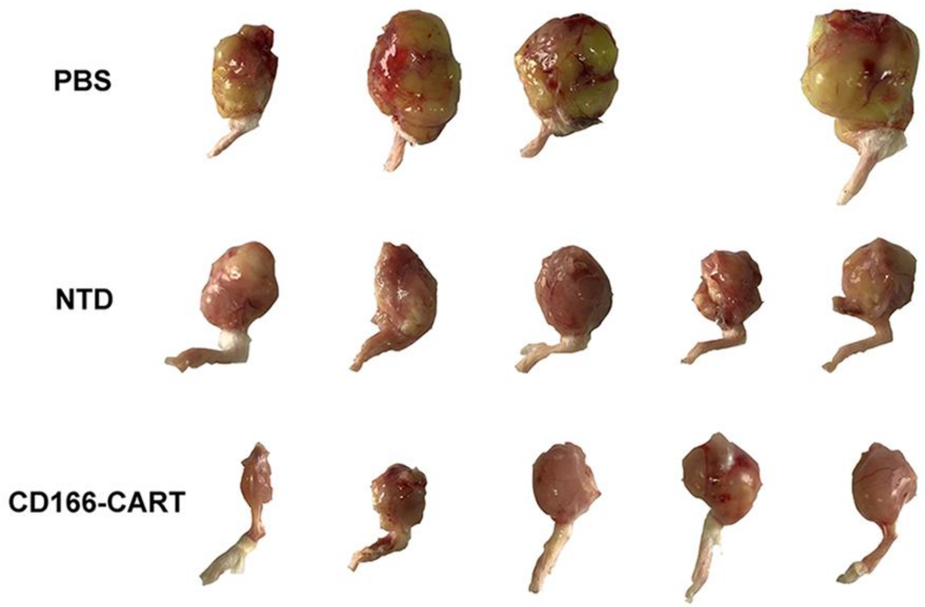

Figure S4. The orthotopic osteosarcoma from different groups were excised and imaged.

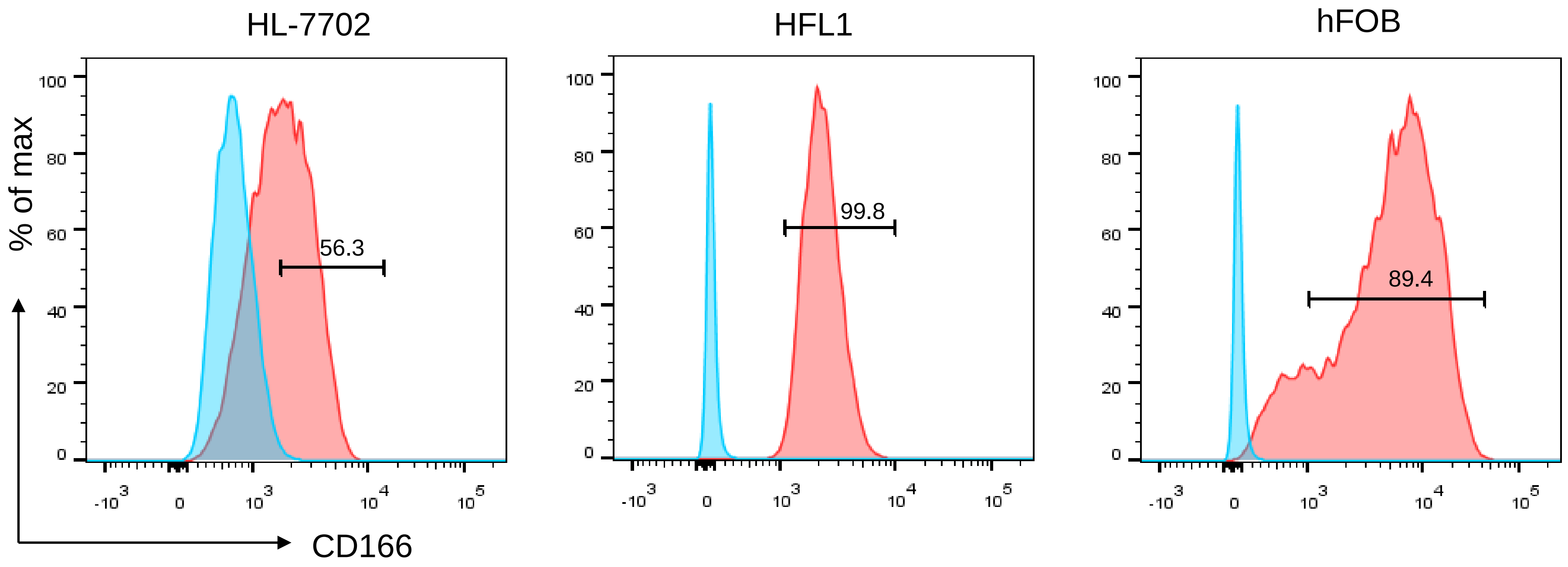

Figure S5. Expression level of CD166 antigen on three normal human cell lines (red histograms). A CD166 isotype antibody was used as a negative control for the detection of CD166 expression (blue histograms). Percentage of positive cells are detailed in the histograms.
